# Advances in Digital Holographic Interferometry

**DOI:** 10.3390/jimaging8070196

**Published:** 2022-07-12

**Authors:** Viktor Petrov, Anastsiya Pogoda, Vladimir Sementin, Alexander Sevryugin, Egor Shalymov, Dmitrii Venediktov, Vladimir Venediktov

**Affiliations:** 1 Faculty of Photonics, National Research University ITMO, 197101 St. Petersburg, Russia; vikpetroff@mail.ru; 2 Faculty of Information and Control Systems, Baltic State Technical University “VOENMEH”, 190005 St. Petersburg, Russia; ap_pogoda@mail.ru (A.P.); sementin.vladimir.v@gmail.com (V.S.); 3 Department of Laser Measurement and Navigation Systems, St. Petersburg Electrotechnical University “LETI”, 197022 St. Petersburg, Russia; sevr_sasha@mail.ru (A.S.); shev1989@yandex.ru (E.S.); dmvened@yandex.ru (D.V.); 4 Faculty of Physics, St. Petersburg State University, 199034 St. Petersburg, Russia

**Keywords:** holographic interferometry, digital holography, spatial resolution, restoring algorithms

## Abstract

Holographic interferometry is a well-established field of science and optical engineering. It has a half-century history of successful implementation as the solution to numerous technical tasks and problems. However, fast progress in digital and computer holography has promoted it to a new level of possibilities and has opened brand new fields of its application. In this review paper, we consider some such new techniques and applications.

## 1. Introduction

**Figure 1:**
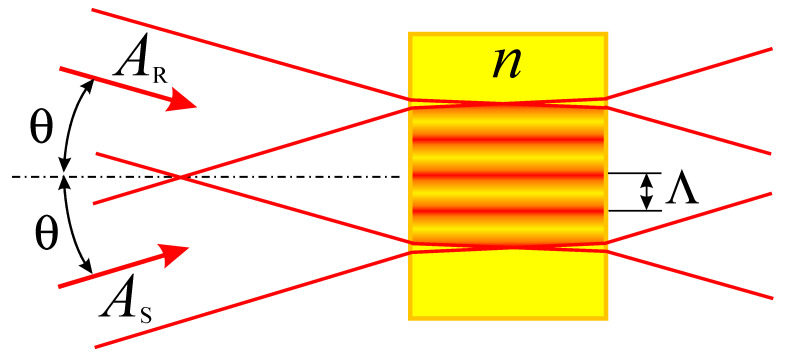
Interference of two plane waves *A*_R_—reference wave and *A*_S_—subject wave in a medium with a refractive index *n*. The period of interference pattern is Λ = λ/2sinθ, where λ—is the wavelength, θ—incidence angle.

Holographic interferometry can be defined as a set of methods for obtaining and interpreting interference patterns formed by various waves, of which at least one is recorded and reconstructed using a hologram. It is a powerful technique of coherent optical measurements for the high-precision analysis of deformations and stresses, sample profile reconstruction, determining the distribution of refractive indices or non-destructive testing. Holographic optical interferometry finds its application in micro- and nanometer measurements [1], mechanical displacements, refractive indices, vibration responses, increasing the resolution of microscopic images, holographic imaging using incoherent illumination, phase retrieval with incoherent illumination, imaging of occluded objects, the holographic recording of depth-extended objects using a frequency-comb laser [2,3], including interference microscopy, quality control and the study of transparent, biological objects, cells and tissues [4], including epithelial ovarian cancer cells [5]. This method is based on recording the result of the interference of two coherent waves—the signal *A*_S_ and the reference *A*_R_ (Figure 1).

**Figure 2:**
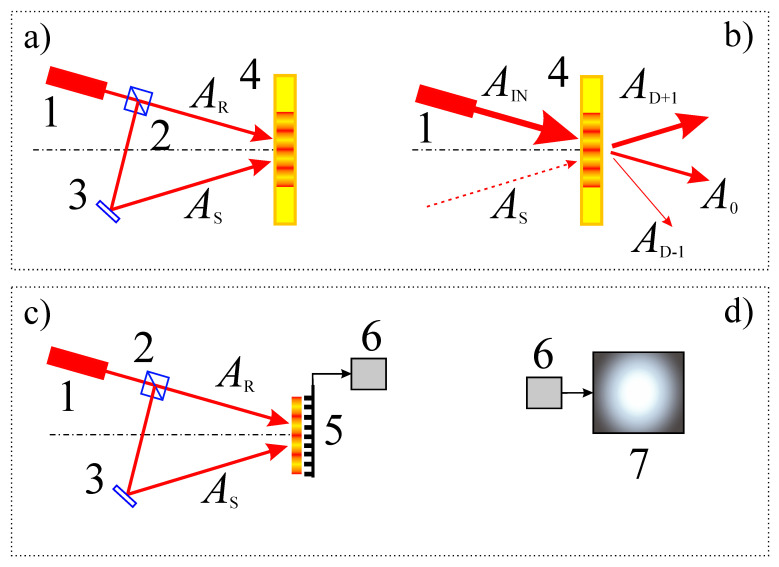
Recording and restoration of a hologram using a photosensitive medium: (**a**,**b**). Recording and digital restoration of a hologram: (**c**,**d**) 1—laser, 2—beamsplitter, 3—mirror (object), 4—photosensitive media (photoplate), 5—CCD camera, 6—processor, 7—monitor. *A*_R_—reference wave, *A*_S_—subjectwave, *A*_IN_—wave before separation, *A*_0_—zeros diffraction order, *A*_D+1_ and *A*_D−1_—first diffraction orders.

Before digital photosensitive devices appeared, holographic interferometry with a recording of an interference pattern on a photosensitive plate was widespread. The spatial resolution of photosensitive plates (up to 5000 mm^−1^) significantly exceeds the spatial resolution of modern digital photosensitive matrices (up to 300 mm^−1^). At the same time, the recording of holograms on a photosensitive plate does not imply automated holographic interferometry (Figure 2a,b).

Holographic interferometry received explosive development after the invention of charge-coupled devices (CCD) with a two-dimensional photosensitive matrix, as well as due to the possibility of using CCD for digital recording of interference patterns in the form of a hologram and the subsequent use of a computer for their numerical reconstructions (Figure 2c,d). Later this method was called digital holography (DH) [6,7,8,9,10].

Digital holography preserves the main principle of «classical» holography—the dependence of the reconstructed image on the phase of the reference beam. However, if in classical holography the human eye is able in this case to perceive the image as three-dimensional, then the image on the monitor screen does not provide information about the volume. Nevertheless, the phase information can be counted and further used, for example, in interferometry.

Usually, four main methods of holographic interferometry are distinguished:

(1) The method of two exposures consists of the recording of two holograms: the object under study before the exposure, which may include deformation, heating, loading of the local area, and the object after such exposure. The recording is made on the same photosensitive plate, and when using digital methods, two arrays of interference intensities of the reference and object waves are recorded on the photosensitive matrix. Hence, the result of interference of fields existing at different timepoints is observed on the plate.
(2) In the real-time method, a scattered wave from an object in its original, undeformed state is registered. Instead of recording a second hologram (registration of a scattered wave from a deformed object), the resulting hologram is restored and the object is illuminated with a single reference beam. As a result, two wavefronts scattered from the object interfere with each other and form a picture of an altered state. Thus, this method allows you to observe the process of deformation of the object.
(3) The time-averaging method is used to estimate periodic loads on an object. In this method, the hologram is exposed over a long period of time, thus averaging the effect of the periodic loading.
(4) The strobogolographic method is similar to the real-time holographic interferometry method. At first, a hologram of an object is recorded in its original, undeformed state, after which periodic fluctuations of the object are provided. During each period of oscillation of the object, it is illuminated by a short light pulse. Thus, this method allows us to estimate the position or state of an object in an arbitrary oscillation phase.

The other existing methods are somehow related to those discussed above.

The rapid development of digital technologies has also contributed to the development of interferometry. Our analysis showed that the methods of recording holograms, interferometer circuits, radiation sources and receivers changed slightly. Here, the progress is mainly associated with the use of multispectral, i.e., multi-wavelength radiation sources. Conversely, the greatest progress over the past decades has been associated with the emergence of new algorithms for processing digital images, including the use of deep learning, which leads to an increase in the quality of recorded holograms and reconstructed objects.

Very conditionally, any digital holographic interferometry system can be represented in the form of the following components: a radiation source, an interferometer and a photodetector with a two-dimensional discrete matrix. After digitalization, the signal from the output of the photodetector enters the computer, where it is further processed. Additionally, the interferometer may have one or more feedback systems for controlling the three previous devices from a computer.

This paper describes the current state of digital holographic interferometry, in particular, the latest solutions in the field of radiation sources suitable for their use in digital holographic systems, shows the current schemes for recording digital holograms, receivers for recording holograms, modern methods of recording and restoring digital holograms, including with the use of deep learning, features filtering, processing and post-processing of holograms and reconstructed objects, as well as the possibilities of applied applications of digital holographic systems.

The review paper is organized as follows. In Section 2, we present some comments on the use of modern radiation sources. A brief overview devoted to some optical schemes used in digital holographic interferometers is presented in Section 3. Section 4 is devoted to the used photodetectors, including the cases of two- and multi-wavelength holographic interferometry and related features. The methods and algorithms for processing, restoring and post-processing of holograms are presented in Section 5. Section 6 provides an overview of the literature related to practical applications. Section 7 contains our conclusions and discussion.

## 2. Radiation Sources and Its Requirements

The irradiation source choice is one of the key tasks that should be solved in the design of digital holographic systems. In particular, to restore three-dimensional objects, one can use a two-wavelength source, two sources with slightly different wavelengths, and a source with a large spectral line width.

In the past five years, methods of usage of a single broadband source have been described. This method is realized due to the simultaneous use of a large number of narrow laser lines at precisely defined optical frequencies. The usage of an interferometer based on two frequency ranges with slightly different repetition frequencies and a camera sensor without a lens allows for recording time-varying spatial interference patterns that generate spectral hypercubes of complex holograms, revealing the amplitudes and phases of scattered wave fields for each frequency of the comb line [3,11]. A similar method is used by other authors [12]. They exploit the high-resolution spectroscopy with the spectra in the form of frequency combs, which simultaneously measure 64 spectra with a resolution of 250 MHz in the 4 nm range during 3 s. When averaging 1000 s, the signal-to-noise ratio reaches 250 per pixel.

Another relevant problem is the design of a digital holographic system for recording and restoring color digital holograms. For example, such a set-up was developed based on a Mach–Zehnder interferometer with a pulse solid-state RGB laser emitting simultaneously at three wavelengths: 451, 532 and 634 nm, which are obtained by converting the irradiation of a pulse YAG:Nd^3+^ laser (1064 nm) operating in the mode of intracavity parametric radiation generation at a wavelength of 1570 nm, and with further conversion of its frequencies into red, green and blue spectral ranges in nonlinear elements from a KTP crystal. The combination of a three-wavelength pulse light source with a Mach–Zehnder interferometer makes it possible to simultaneously register three spectral digital holograms with one RGB matrix radiation receiver [13]. As another interesting work using three wavelengths, we can mention the article [14].

In this work, a color digital holographic interferometry movie was produced by applying the subtraction digital holography method in a quasi-Fourier off-axis experimental setup. The movie was numerically recorded and replayed from three sets of digital holograms obtained with three different laser lines (476 nm, 532 nm, and 647 nm). The movie shows convective flows induced by thermal dissipation in a tank filled with oil.

In recent years, sources of terahertz radiation have become quite widespread. In terahertz digital holography, an off-axis configuration is a suitable choice when the object under study is not sparse and complex. Limiting the recording distance in a non-axial configuration limits the image quality. Potentially, either low resolution or overlapping spectra may occur. An iterative approach to phase reconstruction is used here to improve the quality of reconstruction results obtained from an off-axis hologram. One additional capture of the object’s wave intensity is recorded to perform an iterative phase reconstruction with off-axis reconstruction as an initial assumption. The apodization operation can be applied to capture the wave intensity of an object to suppress undesirable diffraction effects at the boundaries. Using the proposed method, the image quality was improved both during modeling and experimental verification [15].

For quantitative phase imaging in a large range with low noise as a source of radiation, an LED for holographic interferometry can be used [16].

## 3. Overview of Digital Holographic Interferometry Schemes: Schemes Based on Michelson Interferometers, Mach–Zehnder and Other Schemes

This section offers ideas for improving digital holographic systems by adding certain elements to the system.

**Figure 3:**
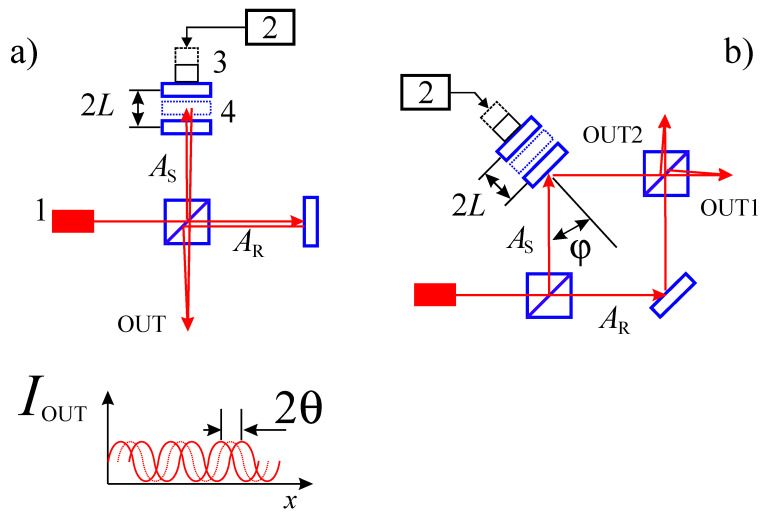
The two most commonly used schemes are the Michelson interferometer (**a**), and the Mach–Zehnder interferometer (**b**), 1—laser, 2—control voltage unit, 3—piezoactuator. The shift of the mirror (4) by an amount of *L* (shift amout) leads to a shift in the interference pattern on θ (phase shift), where θ = (4π*L*/λ)cosφ, *I*_OUT_—dependence intensity on phase, *A*_R_—reference wave, *A*_S_—signal wave.

For example, one of the innovations is the periodical, sinusoidal, or, discrete, stepwise movement of the mirror in the reference arm (Figure 3). The shape of the object is obtained by restoring and processing a sequence of holograms recorded during the stepwise shifting of a mirror in the reference arm [17].

An increase in the spatial resolution of holograms using multiple apertures for recording sub-holograms is also considered. Multi-aperture sampling in the hologram plane allows you to obtain a large hologram field. The sensor’s sparse aperture matrix uses synthetic techniques to achieve a higher spatial resolution than any single aperture that can be used to capture a large-size scene image. However, the accuracy of reconstruction deteriorates significantly in the presence of a large gap between the holes. As a result, the entire scene could not be reconstructed from the hologram field by combining the subholograms [18].

The Mach–Zehnder interferometer circuit (Figure 3b) allows the absolute calibration of the interferometer with high accuracy, i.e., to obtain a relationship between the value of the control voltage on the mirror controller (for example, piezoactuator) and the absolute value of the linear or phase shift of the mirror. For this purpose, a well-proven technique of two-wave mixing is used [19]. It is important to note that this technique allows you to achieve absolute accuracy in determining the periodic linear mechanical displacement of the reflecting surface of the order 0.1–1.0 nm.

Another way to increase the hologram spatial resolution is to use a multi-plane digital holographic algorithm based on extrapolation iterations. The holograms at different axial distances are fixed using a shifting image sensor and then extrapolated to a larger hologram after filling. Multiple holograms provide multipoint constraints when calculating diffraction. Constraints based on the energy distribution and transmission of an object are applied to extract the function of the object from the holograms. The method provides a higher rate of convergence of the inverse problem solution compared to the method of single-plane extrapolation [20].

As a rule, the operation of a holographic system requires high accuracy of adjustment. However, in some cases, the requirements can be reduced. Images recovered from a digital hologram using the Fresnel transformation algorithm at a distance that coincides with the distance from the object to the hologram consist of two parts. One part is a clear image of the object, and the second, caused by the incorrect setting of phase shifts, consists of a set of defocused object images. However, as a result, the quality of the restored image is slightly reduced. Thus, this method makes it possible to abandon the use of a high-precision random phase-shift method [21].

One of the options for complementing the holographic system is to add a light receiver with phase modulation depending on the wavelength. Based on this system, a method is proposed by which multi-wave three-dimensional spatial information is obtained both with a single exposure of a monochrome image sensor and with a sequential holographic multiplexing scheme. This method is applicable not only for laser holography but also for space–time incoherent holography. It was verified that the method works; it was experimentally demonstrated by creating a color multiplexed fluorescent digital holographic microscope and a system of multicolored incoherent digital holography with a white light source [16,22].

Digital holography provides a method of three-dimensional recording and numerical reconstruction using an optical system and a computer. Quantitative measurements and numerical reorientation are the main characteristics. To date, many physical parameters such as amplitude, phase, polarization, fluorescence and spectra can be obtained independently. Recently, multimodal images have appeared, obtained by combining a digital holographic microscope and other optical microscopes, such as a fluorescent optical microscope and a Raman microscope, which allow simultaneously obtaining two or more physical parameters [23].

## 4. Photodetectors with a Two-Dimensional Discrete Matrix

One of the key problems of digital holography is the low spatial resolution of receivers (matrices) compared to photosensitive plates. Thus, one of the tasks that should be solved in the design of digital holographic systems is an increase in the recorded holograms’ spatial resolution. As a rule, most of these methods are based on an algorithm of interpolation of several recorded holograms. For example, one of the methods consists of the spatial shift of the photodetector by an amount less than the value of the spatial resolution element used [24]. There is also a method of numerical interpolation of several holograms by providing a parallel phase shift using a polarizing image sensor [25].

For a more reliable hologram recording, it is necessary to evaluate the recording of sinusoidal signals on the matrix. The author in [26] performed the analysis by direct estimation of the energy incident on each pixel of the photosensitive matrix and took into account the influence of the spatial shift of the signal relative to the array of matrix elements. Thus, it is possible to formulate recommendations for the choice of the matrix photodetector resolution depending on the maximum spatial frequency in the recorded interference pattern.

With two-wavelength holographic interferometry, as in the case of the two-exposure method, a multiplex hologram is recorded. Obviously, in this case, the photodetector should be able to register two interferograms at two different wavelengths.

It is formed from two holograms spatially superimposed on each other and corresponding to different wavelengths. These holograms are characterized by phase distributions (when reflected from an object), where *z* is the depth of the relief of the object; λ_1_ and λ_2_ are the wavelengths used when recording the first and second superimposed holograms, respectively. As a result, due to the variable phase distribution difference of the two superimposed holograms Δφ = φ_1_ – φ_2_, the diffraction structure of the multiplex hologram has a variable contrast, which varies with the spatial period—the beat wavelength Λ:

(1) $$\Lambda =\frac{\lambda_{1}\lambda_{2}}{\left|\lambda_{1}-\lambda_{2}\right|}=\frac{\lambda_{1}\lambda_{2}}{\Delta \lambda }.$$

The beat wavelength Λ is also often called the synthetic wavelength. The range of the measured relief will be limited by the expression [27]:


(2) $$\Delta Z=\frac{\Delta \varphi_{\max}}{4\pi }\Lambda =\frac{\Lambda }{2}.$$

From (1) and (2) it is obvious that the range of the measured relief Δ*z* at close values of λ_1_ and λ_2_ can significantly exceed the limit of λ_1_/2 characteristic of single-wavelength holographic interferometry and reach micro-, milli- or even centimeter range [26]. At the same time, the resolution of the method is not reduced [28]. This method makes it possible to reduce speckle noise, which is especially important in the case of using holographic interferometry to study biological objects [29] and to investigate the dispersion properties of transparent media, which has found wide application in plasma and biology cells diagnostics [30,31].

It is worth noting that with the digital approach, holograms are recorded using a CCD or CMOS matrix, which facilitates the recording of a hologram at two wavelengths and allows it to be produced both simultaneously and sequentially. Moreover, when recording holograms superimposed on each other separately, special techniques can be used to increase the performance of the system: two holograms can be recorded in parallel by using two different cameras or by using different RGB color channel cameras. Then, numerical reconstruction of the hologram is performed and the corresponding distribution of the wavefront phase is calculated, visualizing the measured relief, the dispersion of the medium, etc. In this case, elements of the object under study (for example, relief details) with a length not exceeding can be detected:

(3) $$\Delta x=\lambda_{1}Z/Nl_{\Pi }.$$

where *Z* is the distance between the object and the camera; *N* is the number of camera pixels along *x*; $l_{\Pi }$ is the distance between pixels. As light sources, two separate sources operating at different wavelengths can be used; a source emitting at several wavelengths at once (including a broadband source with bandpass filters) or a tunable source (with sequential recording). With separate recording, the digital approach allows you to operate not only with the phase distribution difference Δφ but also with their sum Σφ. This allows you to switch to another synthetic wavelength:

(4) $$\Sigma \Lambda =\frac{\lambda_{1}\lambda_{2}}{\lambda_{1}+\lambda_{2}}.$$

As a result, the synthetic wavelength used turns out to be less than the original wavelengths (at close values of λ_1_ and λ_2_, the value ΣΛ ≈ 0.5λ_1_). The use of a multiplex hologram reconstructed in ΣΛ makes it possible to increase the measurement accuracy [26,29]. At the same time, an increased measurement range of Δ*z* can also be preserved due to the reconstruction of an additional hologram in Λ [31].

There are several problems associated with the practical implementation of two-wavelength holographic interferometry. For example, there is a double image problem, which consists of the presence of a conjugate wavefront (a double image) during the formation of a hologram, which makes it difficult to reconstruct a multiplex hologram [32]. This problem is solved by hardware modification of the installation and/or software, which is preferable for digital holographic interferometry [27,28,33]. Another problem is caused by the amplification of the noise level in the multiplex hologram: during reconstruction, the phase noise from each of the two spatially superimposed holograms increases by a factor of 2Λ/λ_i_ [34]. Linear regression algorithms [35], the flat field method [36], special neural networks [37] and other methods [35,38] can be used to suppress noise. When using two-wavelength holographic interferometry to measure the phase relief, a problem arises related to the imperfection of the spectral characteristics of the beam splitter, as well as the dispersion of materials and media traversed by the reference and object beams in a holographic installation. As a result, the measured difference in the phase distribution of the two superimposed holograms Δφ is also determined by the dispersion properties of the installation, which leads to an additional systematic error in determining the phase relief. To exclude it, special numerical algorithms are used, for example [39]. It is also obvious that the instability of the difference in the wavelengths used causes an additional measurement error. For example, with the measured relief range Δ*z* = 2 mm, the deviation of the wavelength difference Δλ by 2 pm leads to a relative measurement error of the order of 1% [35].

Two-wavelength holography is a particular type and the most common case of multi-wavelength holography, in which more than two different wavelengths can be used to record or reconstruct one or more multiplex holograms. At the same time, for example, the recording of holograms at three wavelengths with subsequent reconstruction of their multiplex is used to further expand the range of the measured relief Δ*z* [40,41,42,43]. It is also possible to use two frequency comb generators for recording holograms [12]. This allows you to expand the range of the measured relief almost indefinitely without increasing the level of phase noise. Another advantage of this approach is the possibility of multimodal measurements, i.e., the amplitudes of all the holograms reconstructed in λ_i_ can also be used to judge the spectral absorption/transmission coefficient of the object under study.

The two-wavelength operation can be realized by using two lasers with similar wavelengths, one tunable laser, or a laser operating in a multi-wavelength operation mode. An example of such a laser is a solid-state laser based on LiSrAlF_6_Cr^3+^ with the possibility of tuning wavelengths [44,45,46,47,48].

## 5. Methods and Algorithms for Processing and Restoring of Holograms

In this section we will look at the following methods:

- Elimination of zero-order diffraction;
- Digital hologram reconstruction, extraction of the complex amplitude of the object wave in the reconstruction plane;
- Filtering of the holograms and the recovered amplitude of the object wave in the reconstruction plane.

Currently, a special place is occupied by the application of deep learning technologies for the processing and restoring of holograms. Thus, the use of deep learning technologies makes it possible to increase the resolution of the hologram, and determine the recording parameters from the recorded hologram, including a more accurate determination of the distance between the object and the matrix. For example, one of the authors in [49] created a neural network that compares a set of holograms and the corresponding reconstructed images at various scales to study the restoration function.

Additionally, the use of deep learning technologies allows the elimination of the restored image of the 0th order; it compensates for phase aberrations and suppresses noise (for example, with a Kalman filter) [50].

**Figure 4:**
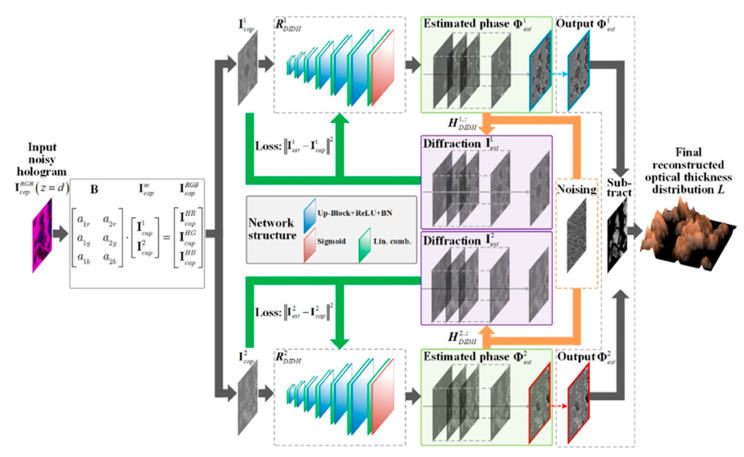
Schematic of the DIDH-Net (Dual-wavelength in-line digital holography) imaging system. A captured hologram $I_{\mathrm{cap}}^{\mathrm{RGB}}\left(z=d\right)$ of a phase object is the input to the neural networks after extracting holograms at each wavelength. The output of the neural networks is taken as the estimated phase${\;\Phi }_{\mathrm{est}}^{m}\left(z=0\right)$, which is then numerically propagated to simulate the diffraction and measurement processes $H_{\mathrm{DIDH}}^{m,z}\left\{\cdot \right\}$ to generate $I_{\mathrm{est}}^{m}\left(z=d\right)$. The mean square errors (MSEs) between $I_{\mathrm{cap}}^{m}\left(z=d\right)\;$ and $I_{\mathrm{est}}^{m}\left(z=d\right)\;$ are measured as the loss value to adjust the neural network parameters. The optical thickness distribution *L* can finally be acquired with the suppressed amplified noises and the free twin-image [51].

Let us discuss recent advances in this topic. The input of the neural network receives a recorded hologram after the selection of holograms at each wavelength. Then, numerical simulation of diffraction occurs at the calculated phase (after passing the channels of the neural network) with subsequent determination of the reconstruction distance. Root-mean-square errors are measured as the loss values for tuning neural network parameters and it is possible to make a depth map that can be obtained by taking into account the use of two phases (passed through a neural network) and noise suppression [36] (Figure 4).

As a rule, neural networks for processing digital holograms are presented on the basis of a convolutional neural network consisting of two main and alternating blocks–convolutional (a hologram convolution operation is performed with a predefined core that can be adjusted and changed) and subsampling (image compression is performed, for example, blocks of 2 × 2 pixels are compressed into 1 × 1 pixels). The passage of images through such blocks trains the neural network by the method of backpropagation. Thus, neural networks solve the problems of hologram filtering and reconstruction and mapping depth [51], as well as eliminating phase aberrations [52].

Convolutional neural networks also solve the problem of determining the reconstruction distance by evaluating the sharpness of the restored image. Thus, the neural network is trained to determine the exact reconstruction distance without direct reconstruction of the holograms [53,54].

**Figure 5:**
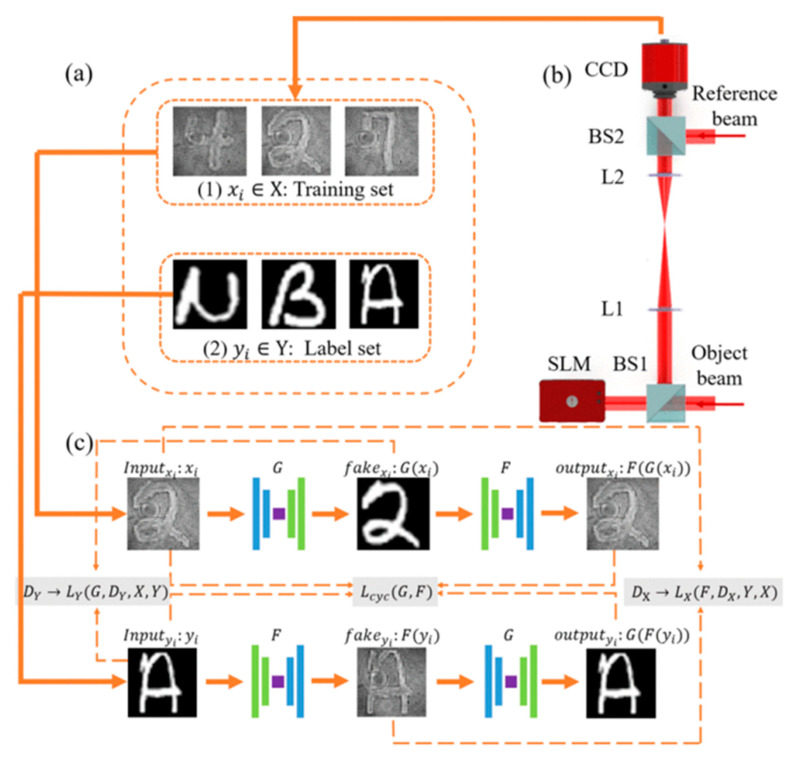
Simplified view of network architecture. (**a**) Training and label set prepared for the network. X_i_∈X: holograms and Y_i_∈Y: labels. (**b**) Partial recording system. (**c**) Overview of training process. Generator function: G and F. D_Y_: images generated by G indistinguishable from real images. D_X_: images generated by F indistinguishable from Holograms. Lcyc (D,F): cycle consistency loss. L_Y_(G,D_Y_,X,Y): Adversarial loss between D_Y_ and G. L_X_(F,D_X_,Y,X): Adversarial loss between D_X_ and F [55].

End-to-end learning-based structures were also developed to reconstruct images without noise in the absence of any paired training data and prior knowledge of the real distribution of objects [55] (Figure 5). The algorithm uses cycle consistency loss and generative adversarial network to implement the unpaired learning method.

It is possible to restore clear images from raw holograms and without preliminary, previously known data, by taking into account the spatial relationship of artificial neurons and embedding information in vectors, not in scalars. Thus, it is possible to overcome the information losses characteristic of convolutional neural networks [56].

**Figure 6:**
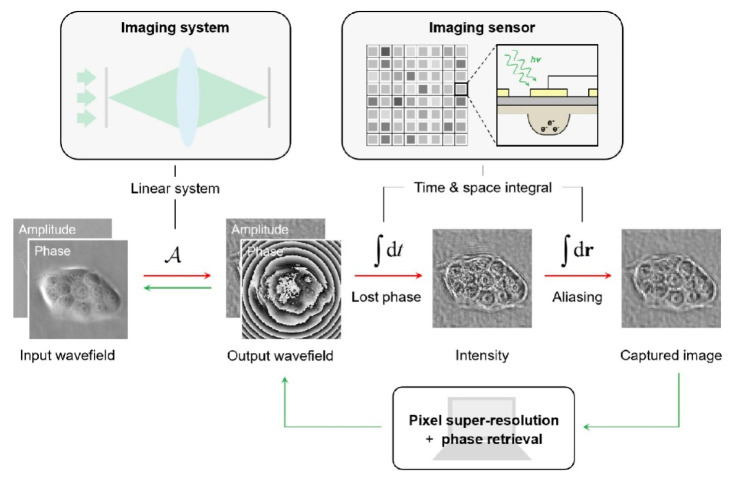
Diagram of the measurement and reconstruction process for in-line digital holography. A—phase difference, ∫dt—time integral, ∫dr—space integral [58].

Another problem solved algorithmically consists of the increase in the hologram resolution. Based on the available data of the Fresnel zone plate model with circular diffraction gratings, the pixel density inside the hologram is increased using the bicubic interpolation method for improvement of the low-frequency components of the object [57]. When using the extrapolation iteration method, higher-order bands are generated outside the hologram to amplify the high-frequency components of the object. The resolution of the reconstructed image can be effectively improved by combining these predicted low-frequency and high-frequency terms. In addition, an increase in resolution is also possible when creating models of transfer functions that take into account the parameters of the restoration of lens-free digital holograms on a chip (close to the receiver) [58] (Figure 6).

Another method of increasing hologram resolution and simultaneously suppressing the zero-order consists of interpolation of the Kronecker product, applied to a single-frame off-axis digital hologram that can generate several spectra of overlapping spectra in its Fourier domain, where the spectra of overlapping zero-order in the extrapolation region can be effectively suppressed. After adding all the regions of the spectrum of the overlapping spectra and replacing each overlapping spectrum in place with their sum, the interception of the spectrum with a larger filtering area can be performed in one of the regions of the spectrum of the overlapping spectra. Experimental results show that the method is suitable for improving the resolution of off-axis digital holography [59].

The image quality of the embedded digital holography is reduced by the effects of double imaging and overlapping spectra since the sensors respond only to intensity, and the pixels have a finite size. As a result, the methods of phase extraction and ultra-high pixel resolution serve as two important components for creating holographic images with high accuracy and high resolution. Thus, these two problems could be combined into a single optimization issue. This method is implemented using an iterative projection algorithm and a gradient descent algorithm [60].

Let us consider some algorithms and methods of filtering holograms. For example, some authors use the following method: the recorded field is perceived as subdiscretized, filtered out using a low-pass filter and projected based on Fresnel–Bluestein in the inverse problem approach to image reconstruction with controlled lens-free magnification [61]. Another author uses advanced digital filtering to eliminate the effect of a virtual image; three-dimensional volumetric deconvolution to reduce the problem of depth of field [62].

Another problem is the suppression of speckle noise in digital holography without distorting the reconstructed image. A solution is proposed in the form of using a combination of the Grab-Cut algorithm–the selection of an object from the background–with a controlled filtering algorithm. The result is a quasi-noiseless reconstructed image [63].

The reconstruction of digital holograms requires evaluation of isoplanatic phase errors that do not work well in the presence of noise or large phase errors. It is proposed to use an iterative reconstruction algorithm based on a model that calculates the maximum aposteriori estimation of the phase and reflection coefficient of an object without speckles. The algorithm is also resistant to high noise and significant phase errors [64].

The process of modeling the hologram recording is also considered. Thus, by accurate and numerical modeling of the propagation of a coherent light source through a number of optical elements and the object itself, it is possible to predict the optical interference of the object and the reference wave in the recording plane, including the effects of diffraction, aberration and speckles. It is shown that the optical transformation predicting the complex field in the recording plane can be generalized for arbitrary configurations of holographic records using the matrix method. It is also possible to present a description of digital phase reconstruction and aberration compensation for various off-axis holographic configurations [65].

As a rule, the hologram reconstruction is performed by calculating the Fresnel–Kirchhoff integral based on the Fourier transform. However, one author used another algorithm, namely, solving an optimization problem in which the minimized loss function consists of a data approximation term in the L2 metric and modified penalty methods of the Huber function, which are alternately minimized in an adaptive way. The author considers this method to be more accurate than the Fourier transform [66].

**Figure 7:**
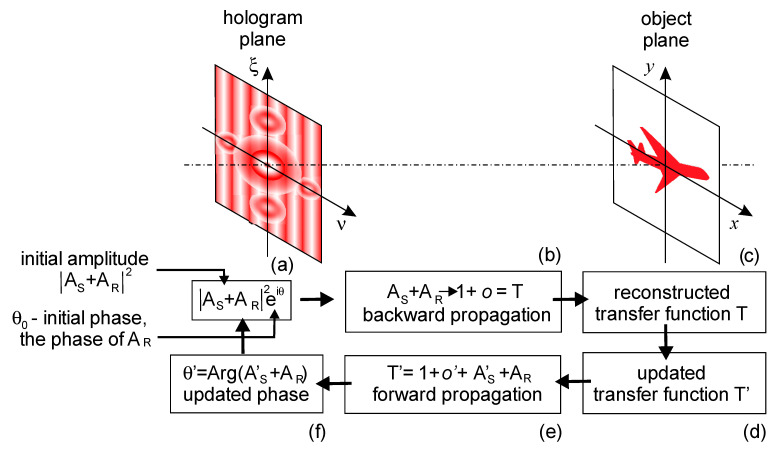
Explanation of the iterative phase retrieval algorithm from a single-shot intensity measurement. The algorithm starts in the plane of the hologram. The hologram showed conditionally (**a**). The initial complex-valued distribution is created by combining the measured amplitude distribution with the phase of the reference wave. *A*_R_—reference wave and *A*_S_—signal wave, θ—phase. The wavefront propagates from the hologram plane to the sample plane, where it gives the distribution complex-valued transfer function *T*(*x*,*y*) (**b**). Restrictions in the sample plane are applied, and the updated transmission function *T*″(*x*,*y*) is obtained (**c**,**d**). Forward propagation (**e**): the wavefront propagates from the object plane to the hologram plane, where the two-dimensional detector (**f**) is located. The amplitude of the wavefront distribution in the hologram plane is replaced with the measured amplitude. The complex-valued wavefront distribution in the detector plane is updated for the next iteration starting at (**a**).

An alternative method for reconstructing the object wave phase is based on the Gerchberg–Saxton algorithm. The algorithm allows reconstructing three-dimensional samples by reconstructing a complex-valued wavefront using reciprocating propagation of the wavefront between two planes with constraints imposed on these two planes. Interactive phase reconstruction allows for restoring the amplitude and phase distributions of an object quantitatively correctly and without double images from its embedded hologram. In this method, reconstruction is performed by applying iterative phase reconstruction between the planes where the intensity was measured (Figure 7). The reconstructed complex wavefront then propagates back to the sample planes, thus reconstructing the three-dimensional distribution of the sample. This method can be applied to three-dimensional samples, such as three-dimensional particle distribution, thick biological samples and other three-dimensional phase objects [67].

The hologram reconstruction process is a Hermitian transformation with respect to the hologram formation process. Based on the properties of the Hermitian transformation, it is possible to determine the function of a three-dimensional object. This method is an alternative to the standard method, for which it was considered that the hologram reconstruction is the inverse transformation of the hologram formation process [68].

An algorithm for hologram reconstruction using phase-structured lighting and a single-pixel sensor also exists. The object is measured using a set of microstructured phase patterns implemented on a liquid crystal spatial light modulator, while a single-pixel sensor sequentially registers fluctuations in illumination corresponding to interference between the object and the reference rays. The reconstruction algorithm extracts unknown phase information from a complete set of photocurrent measurements. The sampling functions can be encoded on the reference beam, so they can be non-local with respect to the object. The system is also well adapted for transmitting information about an object through scattering media [69].

When reconstructing digital holograms, wavelet transformations can be used, in particular, Cohen–Daubechies Fe 9/7 and 17/11 wavelets for lossy data compression [70,71].

Thus, in this chapter, modern algorithms for reconstruction, filtering and processing of holograms were considered.

The use of digital holographic systems implies the transmission of data arrays through communication channels. Algorithms for filtering and compressing holograms are used to increase the throughput of digital holographic systems.

Binarization of digital holograms is used for storage and operational transmission of recorded information, as well as for optical image reconstruction using binary micro-mirror light modulators. There is a possibility of binarization of optically registered holograms by iterative methods using the error diffusion operation at each step of changing the binarization threshold or only at the final iteration. Due to the binarization of holograms using the error diffusion operation only at the final iteration the quality of image reconstruction increases by 12% compared to one-step methods [72,73].

Reconstruction and filtering of high-resolution holograms are resource-intensive tasks that require high information throughput of computer channels for transmitting digital holograms with a resolution of the order of tens of gigapixels. To reduce the load on the computer, it is proposed to create and reconstruct horizontal parallax digital holograms [74].

## 6. Digital Holography Applications

Historically, digital holography began to receive its first practical applications in industry. High-precision analysis of mechanical vibrations, mechanical deformations, and stress tests—all these tasks were solved by holographic methods [75,76,77].

The advantages of digital holography make it possible to measure temperature in liquid flows with high accuracy [78].

**Figure 8:**
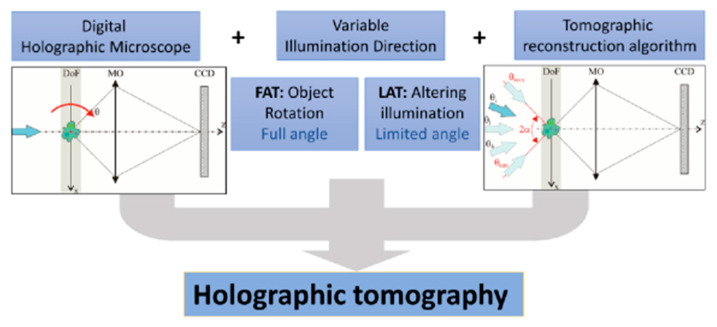
Basic modules of holographic tomography: FAT, full-angle tomography; LAT, limited-angle tomography [4].

Digital holographic volumetric reconstruction usually uses multiple diffraction calculations to obtain sectional reconstructed images from an embedded hologram, followed by the determination of lateral and axial positions, as well as particle sizes using focal metrics (Figure 8). The speed of such a system can be increased by using a deep neural network [79]. It is also possible to recognize particles, and determine their positions and sizes using clustering algorithms with the recognition of particle edges and background [80].

The generation of three-dimensional structures of microparticles by means of digital holography applying the automatic focusing method is also used. This is carried out by superimposing a set of silhouette-like particle images reconstructed from a single embedded hologram. The method provides estimations of the particle size in longitudinal and transverse dimensions. Using the discrete dipole approximation, the method is verified computationally by simulating holograms for various particles and attempting to reconstruct known three-dimensional structures. The low longitudinal resolution strongly perturbs the reconstructed structure, however, the method gives an approximate estimation of the longitudinal size of the structure [81].

In the work [82], the authors consider the method of three-dimensional quantitative phase imaging which provides the 3D distribution of the refractive index and the dry mass in live and fixed cells as well as in tissues.

**Figure 9:**
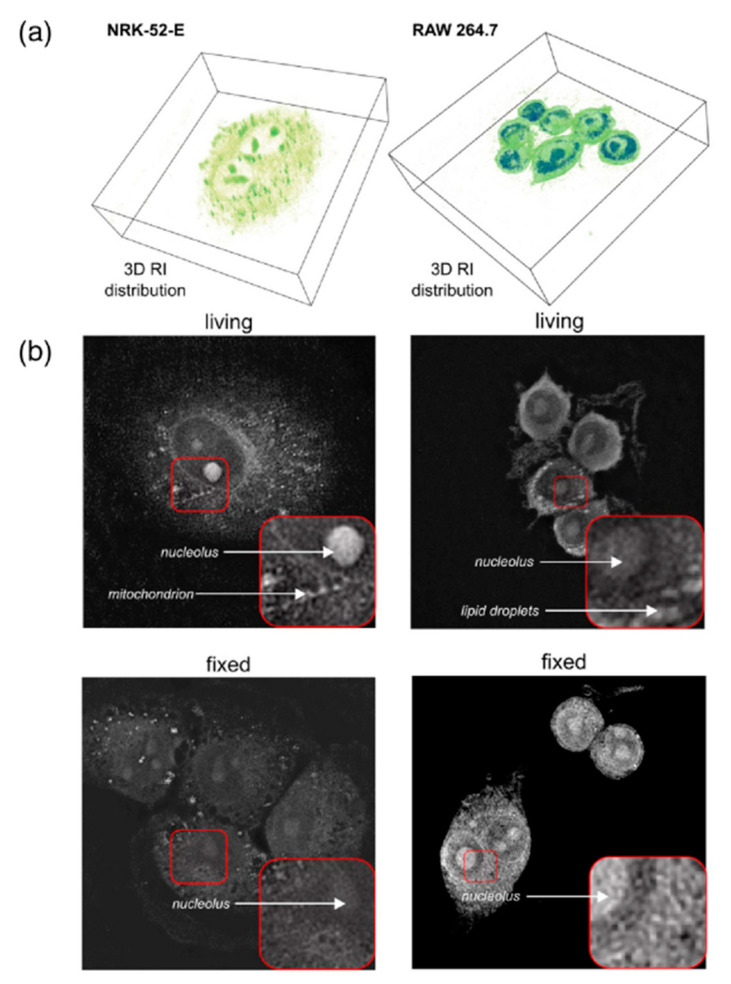
The holographic tomography measurements and analysis of four cell lines: (**a**) 3D visualization of cell lines NRK-52E and RAW 264.7, (**b**) 2D refractive index distribution cross-section of the RI distribution in the best focal plane of live and fixed NRK-52E and RAW 264.7 cells [4].

In [83], an algorithm for transparent object reconstruction is suggested. The image reconstructed using this method expresses the phase distribution of the light wave after passing through the object under study, since the phase change inside the object is difficult to determine (Figure 9). Then, the Abel transform is applied to a high-speed phase image of a dynamic transparent object, which is assumed to be axially symmetric. The phase is accurately recorded by the phase shift method. Thus, it is possible to experimentally fix a transparent dynamic gas flow, assumed to be axisymmetric with respect to the direction of the gas flow, at a speed of 3000 frames/s and to restore the three-dimensional distribution of the refractive index of the gas from a high-speed motion-picture phase imaging obtained by a parallel phase, in other words, to restore the dynamic process.

The effectiveness of using the inverse Abel transform for digital holography problems was demonstrated in [84]. The technique was developed based on using fringe contouring, polynomial phase fitting and inverse Abel transformation, to reconstruct the three-dimensional dose distribution in noisy conditions. In order to assess the feasibility of this approach in measuring the absorbed dose distributions in noisy conditions, the whole approach is modeled for high-energy electrons. It is shown that the three-dimensional dose distribution inside the absorbing medium can be obtained with good accuracy even in the presence of considerable amounts of deliberately added noise to the holograms.

Digital holography methods are also applied in the industry. For example, in [85], a hardware and software complex of flaw detection for the determination of the geometric dimensions of defects on aviation glasses samples was developed.

Among the applications of digital holography in the industry, the topography of the surface should be mentioned. It could be realized by the method of frequency conversion, which is based on the principles of interferometry with scanning along the wavelength. The method allows measuring the surface relief of objects with a diameter of several hundred mm with a high axial accuracy reaching 10 microns. The main advantage of the system is the fact that the surface topology data are fixed immobile using a relatively simple installation [86]. Thus, the method can be used for topographic measurements of objects of complex geometry made of conventional materials (such as metals, plastics, etc.), as well as for the characterization of complex composite structures, such as acoustic metamaterials, active acoustic metasurfaces, etc.

Digital methods can be also used for modification of the hologram structure. One can, for instance, carry out the so-called amplification of the interferogram, i.e., increase or decrease in its spatial carrier frequency, which can be sometimes useful. In addition, in nanointerferometery it is sometimes necessary to eliminate the impact of distortions, imposed by the elements of the interferogram. In the pre-digital epoch, these procedures required re-recording of the interferograms per their summation. The authors of this review have carried out the cycle of research activities on this subject, which were summarized in the paper [26].

In conclusion of this review, we would like to note the most impressive achievement related to digital holographic interferometry. The combination of digital holography phase-contrast microscopy methods led to the creation of a unique technology—holographic tomography [4,87,88,89]. This technology allows you to simultaneously study the thickness of the object (absorption) and the refractive index. For example, the authors of [86] managed to develop a device combining sample rotation and illumination, which allows obtaining images with an isotropic resolution below 200 nm. Such digital holographic devices allow you to create various methods for quantitative microscopic imaging.

They are quantitative imaging of single cells and their internal organelles; monitoring of cell processes and pathology; and investigations of tissues and small-scale biological objects [90,91]. It should be especially noted that this direction has confidently moved from scientific developments to the commercial implementation of industrial devices [92]. It can be concluded that the use of optical, holographic tomography has moved from optical laboratories to the walls of medical laboratories [90,93].

## 7. Conclusions

The wavefront recording technique already proposed by Leith E. N. and Upatnieks J. back in 1964 and called “holography” has made impressive progress in the current century. The prerequisite for this progress was the appearance of high-quality two-dimensional photodetectors and high-speed computers. This made it possible to perform various mathematical operations on an array of discrete data describing the result of the interference of two waves, i.e., a hologram. Now it is possible to restore the original object in a digital form. As a result, we have exciting opportunities due to the emergence of algorithms that can be classified by the general term “artificial intelligence”. The new technical direction, called Holographic Tomography, combines the advantages of optical holography, phase microscopy, digital tomography and artificial intelligence. The images that allow us to examine in detail the behavior of living biological objects, including cells, are truly impressive. The images obtained with the help of Holographic Tomography allow you to observe the processes in living tissues in real-time and in great detail. This makes these technologies a powerful tool in the detection and treatment of various clinical diseases.

It should be noted that the reader can find some more fairly new reviews, see for example [94,95]. These reviews are much more extensive and cover a large number of application areas. We tried to focus on biomedical applications, which is part of our interests.
